# Supplementary material for: Locally Prepared Therapeutic Food for Treatment of Severely Underweight Children in Rural India: An Interventional Prospective Controlled Community-Based Study with Long Follow-Up:—‘SAMMAN’ Trial
Source: Nutrients. 2024 Aug 27;16(17):2872. doi: 10.3390/nu16172872 (PMC11397721; doi:10.3390/nu16172872)
Supplement: Supplementary file 1 [file nutrients-16-02872-s001.zip › nutrients-3103363-supplementary.pdf]

Supplementary Materials

File S1. Study area and Sample size.

1. Study Area

This study was done in Melghat region of Amaravati district in Maharashtra, India. This is difficult to access, hilly, forest, tribal area (>4000 square kilometer) in the Satpuda mountain ranges. It includes 320 villages with a population around 3,00,000, around 85% of the population are tribal. Most of the tribal (>90 %) are small farmers with around one hectare land or laborer and live below poverty line.

Most of the villages have poor access to roads, transportation, electricity, education and health care. This area is known for very high prevalence of malnutrition i.e. 22% of severe malnutrition (2011) and high under 5 children mortality rate (U5MR) (>100 per 1000 live births) especially malnutrition related deaths. The health care is provided by the Government facilities and private practitioners. The average distance of a village from a MBBS doctor, medical shop and a district hospital is 19, 28 and 160 kilometers respectively. The primary health care delivered by the Government is provided by paramedic workers and a primary health care centers (PHCs). A single paramedic worker covers a population of 5000 and one PHC with two physicians provides health care to a population of 20,000. There are total 11 primary health centers, 2 rural hospitals & one sub-district hospital in Melghat region. The infant and child health-care programs implemented by the Government in this area include integrated child development schemes for food supplementation by Self Help Groups (SHGs) and weight record of children, immunization, de-worming and distribution of oral rehydration solution (ORS) is done by the paramedic workers. However, health Care-seeking from tribal, is low. Most of the tribal prefer to go to the traditional faith healers for treatment.

2. Selection of sample size from the study area

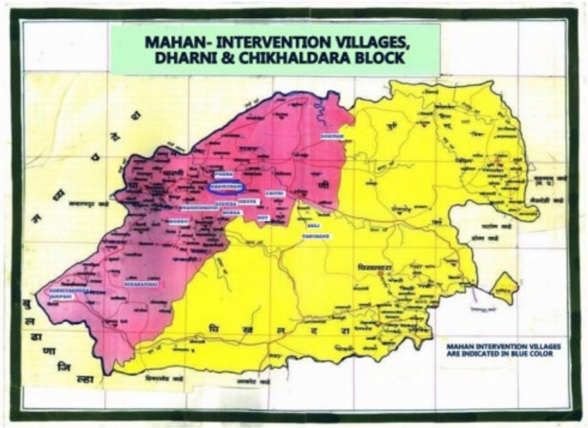

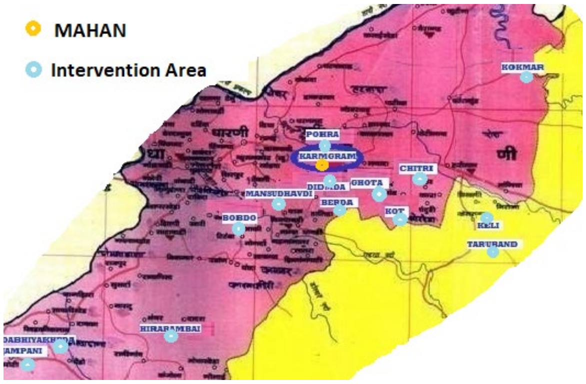

Melghat, a rural -tribal/ area in Maharashtra, India, consisting of 320 villages(clusters), divided in Dharni and Chikhaldara blocks with a population of approximately 3,00,000, of which 84 % are tribals. The Dharni block was divided in five zones, after stratification, based on distance from base hospital, three clusters from each zone were selected. One cluster dropped out due to unwillingness for participation. Thus 14 representative study villages were randomly selected (Appendix 1). These villages represent the demographic, socioeconomic characteristics, health indicators, and malnutrition (SUW) of maximum Melghat villages. These villages are Kokmar, Chitri, Dabhiyakheda, Jampani, Berdaballa, Didamda, Ghota, Mansudhavdi, Hirabambai, Bothara, Pohara, Keli, Kot and Tarubanda.

The study participants included the 'IA' which consisted of SUW children aged 6–60 months and the 'RA', which consisted of age-matched (±two weeks) control children with normal WAZ (non-SUW), from the same demographic study area, whose parents gave informed written consent and were permanent residents of the study area. Children who migrated from the study area for more than six months were excluded from the study.

The purpose of adding comparative reference arm (RA) of normal children was to know whether the impact of our intervention on recovery, growth pattern, BMI, and case fatality rate of SUW children is similar to, non-SUW normal children at the age of 60 months. So that the treated SUW children in IA would have similar outcome indicators to those of normal children in RA. Our ethical committee did not permit, diagnosing SUW child and yet not treating them, as that would be unethical. Hence, we included normal, non-SUW children as RA.

The children in IA and RA were selected from same 14 study villages. So, it was not possible to calculate the impact on prevalence due to our intervention. Thus, to compare and analyze impact of our interventions on SUW prevalence in the 14 study villages, another group of 22 control villages was randomly selected.

We conducted our intervention in this study at the village level and therefore those villages are named as intervention villages. In the intervention villages, we targeted severely underweight

children (SUW) as intervention arm (IA) for treatment by LTF-MN to know its impact. In the same intervention villages, we selected reference arm (R.A.)- non-SUW children, age-matched (±two weeks) as controls.

Supplementary File S2. Intervention planning and details of intervention

The survey was conducted from September 2010 to December 2010 revealed very high prevalence of SUW (21.5%), faulty socio-cultural feeding practices, child care practices and poor hygiene. Participatory community meetings provided introduction of the study to the community, their collaboration and identified resources for child health and potential barriers to implementation and factors affecting behavior change and low health-care seeking behavior.

Preparatory phase (January 2011-April 2011):

a) Intervention planning/ Microplanning: The community-based interventions were planned based on qualitative focus group discussions and survey findings in accordance with communities' culture, traditions, language, impoverished status, demands and low health seeking behavior with public patient involvement (PPI).
b) Informed written consent of the community in each cluster was obtained through Gramsabha/community-meetings and from parents of all study children. The condition of the gramsabha was that at least 60% of the people above the age of 18years must be present in the meeting and more than 60% must give permission to conduct the study.
c) Selection and training of team was done (Appendix 3)
d) The demographic and socioeconomic characteristics were used from a MAHAN survey 2009–2010. (Supplementary appendix 4)
e) SAMMAN questionnaire form was designed. Attached.

Intervention phase (May 2011 to Dec 2019)

Enrollment: The enrolment of SUW children initiated in the month of May 2011 and continued till May 2015. All the children were given local therapeutic food (LTF) + micronutrients (MN) nutrition therapy for 3-months. The LTF intervention was stopped in August 2015. The last enrolled case was followed up till October 2023. Age matched controls of normal children with same demographic characteristics were selected from same study area.

VHWs recorded monthly anthropometry (weight, height and mid upper arm circumference) of all children aged 6-60 months from study area. Standardized digital weighing machines calibrated to 5 gms and standardized stadiometers/infantometers calibrated to 0.5 cm were used for weight and height/length record respectively. The data was transferred to the data section. Customized software MAHAN-soft, version 1.0, 2011, based on WHO Child Growth Standard (2007) WAZ for gradation of malnutrition in the base research center of MAHAN. The data manager prepares the list of SUW and give to medical supervisor (MS). MS cross-checked anthropometry of all SUW children and prepared final list of SUW for enrollment in study with VHW.

Intervention in the community

1. LTF MN Therapy:
    - Appetite test (1) (Supplementary appendix 5)
    - LTF-MN feeding for 90 days (Supplementary appendix 6)
2. Behavior change communication (BCC) was done by BCC supervisors and VHW's. (Supplementary appendix 7 and 8)
3. Medications administered for treatment of infections by VHW's (2) (Supplementary Appendix 9)

- **IA received LTF-MN therapy, Intensive BCC and treatment of infections by VHW.**
- **CA received only BCC and treatment of infections by VHWs.**

4. Yearly point prevalence of SUW was determined from 2011 to 2019 in Aug-Sept.

References

1. Michael H. Golden YG. Guidelines for the management of severely malnourished: WHO and UNICEF; 2010. 27-8 p.
2. Handbook : IMCI integrated management of childhood illness. Geneva: World Health Organisation; 2005.

Supplementary File S1: Selection, training and monitoring of team

The study team consisted of- VHWs, supervisors of three categories (i.e. medical supervisor, BCC supervisors, data collection supervisors), retrospective surveyors, program manager, pediatrician and project director.

A. Selection of team

1. Village health workers' (VHW) Selection

- Eligibility criteria:
    - Female
    - Semiliterate: Education at least 5th standard.
    - Should belong to same village and community and should be able to communicate in local dialect /language.
    - Married so that she can continue for a longer period. Preferably she should have a child.
    - Should be sensitive to the problems of society.
    - Must be selected by the community i.e. acceptable to the community so that it will empower community participation. Community engagement led to selection of appropriate candidate.

- Selection Process

  Pre planned village meetings (gramsabha) were organized in each village. Village head, police patil and at least 60% of all the adults (both males and females) of the village were present. Information about the project was given. The need and eligibility criteria for VHW were explained. Community participation was vital for the project.

  Three women were selected by the gram-sabha (community meetings) and called at MAHAN base hospital for four-day residential selection camp. Detailed information about the project was given e.g. role of the VHW, expectation of the work from VHW, incentive for work, award-punishment process. They were given a poster and asked to speak about it. To check the attitude & self-interest of person, we organized shramdan (labour donation) and allotted them responsibilities e.g., food committee, care committee, cleanliness committee and a committee to observe the participation of every woman. They were sent to few villages for field testing to know how they interact with the community. Communication skills were closely monitored. W ritten examination and oral interview was conducted. At the end of camp, one VHW was selected from each village.

2. Selection of other staff

Supervisors and program manager were selected through theory examination, oral interview and field visit observations.

Supervisors were of three types:

a. Medical supervisors
b. BCC supervisors
c. Data collection supervisors.

B. <u>Training of team</u>

1. Training of the VHWs

Monthly initial residential trainings of 5 days each were conducted for one year. Then refresher trainings were conducted 3 days every month for one year. Afterwards, 3 days trainings were conducted every alternate month till the end ofthe project.

A module, set of slides and audiovisual flip charts were developed for Community based management of SUW children and specific form developed for the project.

1. How to record anthropometry of children
2. VHW trained for feeding of LTF-MN in appropriate amount as per WHO guidelines
3. Intensive BCC (Supplementary appendix 8) was given to parents of children from IA
4. BCC was given to parents of children from CA (Supplementary appendix 7)
5. Administration of medications for the following diseases: ARI, diarrhea, episode of fever and malaria. (Supplementary Appendix 9)

Method of trainings: Class room training and field training, lecture, group discussion, demonstration, participatory learning, peer group learning, pictorial flipchart, audio- visual show, role play, etc.

The supervisors were responsible for field trainings of VHWs, providing support and guidance to them in the field.

Training of other staff

Supervisors and program manager were trained through MAHAN modules and field trainings by consultant pediatrician and program in-charge.

C. Monitoring of field staff:

1. Monitoring of VHWs:

In the field: The treatment of each patient given by 5 to 6 VHWs were monitored once a week by one medical supervisor and BCC of parents by 5 to 6 VHWs were supervised weekly by one BCC supervisor. Vital statistics data collection of 10 VHWs were monitored fortnightly by each data collection supervisor. The project manager monitored the activities of each VHW once a month.

From the record: The VHW activities were monitored regularly by checking their various forms and registers by supervisors (once a week) and project manager (once a month). The pediatrician and project in-charge monitored the records once a month.

Meetings: The pediatrician and project in-charge conducted weekly meeting of all supervisors and program managers and monitor their work. They also conducted monthly meetings with VHWs to monitor their work.

2. Monitoring of the supervisor:

The daily activities of all supervisors were monitored through daily report by the project manager & through weekly/ monthly meetings and reports by the pediatrician and project director. The project manager monitored the supervisor activities during field visit once a month.

3. Monitoring of the project manager:

The project director and Pediatrician monitored the activities of project manager via daily report, during weekly meeting & monthly report.

4. **Project director and pediatrician** visited the villages to monitor activities of all subordinate staff intermittently and whenever needed.

Supplementary File S2: Demographic and Socioeconomic Survey

As per 2009 CENSUS done by MAHAN Cross check census

Sr. No.	Indicators		No.
**Usual Resident Population**			
1.	No. of villages		14
2.	Total population		13038
3.	Crude Birth Rate		26.2
4.	6 to 60 months age group population- n (%)		1434 (11%)
5.	Tribal U5 children population		1305 (91.0%)
6.	Average Family Size		6
**6 to 60 months age group Population (N= 1434)**			
7.	Gender- Male: Female		1.06:1, (739/695)
8.	Age Group	6 to 24 months	614 (42.8%)
		25-60 months	820 (57.2%)
9.	Maternal literacy	Illiterate	674 (47.0%)
		1st to 4th Std.	314 (22.0%)
		5th to 10th Std.	409 (28.5%)
		11th to Graduate	37 (2.5%)
10.	Paternal literacy	Illiterate	274 (19.1%)
		1st to 4th Std.	367 (25.6%)
		5th to 10th Std.	672 (46.9%)
		11th to Graduate	121 (8.4%)
11.	Open defecation		1428 (99.6%)
12.	Firewood Cooking		1333 (93.0%)
13.	House pattern (n=1277)	Roof of thatch	685 (47.8%)
		Roof of Tin	350 (24.4%)
		Roof of Kavelu	268 (18.7%)
		Roof of cement	5 (0.3%)
14.	Agricultural land*	No land	834 (58.2%)
		1-5 Acres	466 (32.5%)
		> 5 Acres	134 (9.3%)

** vast majority had non-irrigated agricultural land (>80%)*

Supplementary File S3: Appetite test by MAHAN

Need for the Appetite test:
Presence or absence of good appetite in the malnourished child is very important to plan the nutritional therapy of a child. Children with good appetite can be treated at home settings with the help of VHWs. Children who have low appetite have to refer to the higher health care centers. Therefore appetite test becomes important tool to check the appetite of a child before enrolling to the local therapeutic food (LTF)-micronutrient (MN) therapy.
Indication: All severely underweight (SUW) children (Weight-for-Age <-3SD) from intervention area (IA) are examined for Appetite test.
Place: at feeding center situated at VHWs house administered by VHW under the supervision of Medical supervisor.
Food to be used in Appetite Test: Any of the LTF-MN preparations of childs liking (Appendix 6). We also offered F-75 and F-100 to test appetite test in the beginning of the study for SUW with complications.
Method: Mother is explained about the appetite test. After washing hands with soap and water, child is comfortably placed in mothers lap.
The LTF preparation is given to child according to body weight:

**Body Weight**	**< 5 kg**	**5-9 kg**	**> 9 kg**
LTF-MN Packet	1/4th packet (25 gm)	1/2 packet (50 gm)	Full packet (100 gm)
K Calories	137.5	275	550
Proteins (gms)	3.25	6.5	13

Interpretation:

**Food consumed by the child**	**Result**
More than half/complete	Pass
Half	Intermediate
Less than half	Fail

If failed-
Child is referred to hospital for check-up.
If parents are not willing for hospital check-ups, treatment is given by Village Health Workers (VHW) under supervision of Medical Supervisors (MS) after high risk consent by parents. LTF-MN therapy was started.

Supplementary File S4: Preparations of "MAHAN" Local Therapeutic Food and Minerals (LTF-MN))

MAHAN LTFs are an appropriate source of therapeutic food that provides complete nourishment to the SUW children. There are eight varieties of LTFs and SUW children can choose from them as per their choice. All the LTFs were hygienically made local foods specifically prepared by local people who were trained under the vigilance of experts. Each ingredient's nutritional value was calculated and externally verified through laboratory analysis.
All the LTFs were designed to meet the WHO guidelines for the treatment of severe acute malnutrition (SAM).
The preparations are multiple, palatable and socio-culturally acceptable therapeutic food for malnourished children and can be used in other tribal blocks of India.

Supplementary Table 1 (S1) : Local Therapeutic Food (LTF)- Composition and macronutrients

Sr. No.	LTF	Ingredients	Amount	Protein	oil	energy	Remarks
			Gram	Gram	Gram	Calories	
A. Ready to consume LTF (24-59 months):							
A.1	Sesame jaggery groundnut daaliya chikki	Sesame	10	1.83	4.33	56.3	Ready to eat
		Jaggery	25	0.1	0.025	95.8	
		Groundnut	30	7.59	11.94	170.1	
		Daaliya	20	4.5	1.04	73.8	
		Soya oil	15	0	15	135	
		Total	100	14.02	32.335	531	
A.2	Chiwada	Groundnut	30	7.59	11.94	170.1	Ready to eat
		Daliya	30	6.75	1.56	110.7	
		Pohe	10	0.66	0.12	34.6	
		Murmure	10	0.7035	0.01	32.5	
		Soya oil	20	0	18.76	180	
		Total	100	14.02	32.335	531	
A.3	Jaggery groundnut Chikki	Jaggery	35	0.14	0.04	134.05	Ready to eat
		Groundnut	55	13.92	21.89	311.85	
		Soya oil	10	0	10	90	
		Total	100	15.70	32.39	527.9	
B. Powdered ready to use snacks for 6-months to 24-months age group							
B.1	Groundnut jaggery powder	Jaggery	35	0.14	0.04	134.05	Ready to eat
		Groundnut	55	13.92	21.89	311.85	
		Soya oil	10	0	10	90	
		Total	100	14.06	31.93	535.9	
B.2	Pohe daaliya powder	Pohe	20	1.98	0.03	97.5	Ready to eat
		Daaliya	30	4.5	1.04	73.8	
		Groundnut	30	7.59	11.94	170.1	
		Soya oil	20	0	20	180	
		Total	100	14.07	33.01	521.4	
C. LTF to be cooked (6-59 months)							
C.1	Thalipeeth/ upma	Jawar	20	2.08	0.38	69.8	Cook before serving
		Groundnut	30	7.59	11.94	170.1	

		Chana dal (besan)	30	6.24	1.68	111.6	/Ready to heat.
		Soya oil	20	0	20	180	
		Total	100	15.91	34	531.5	
C.2	Moong khichdi	Moong dal	25	6.1	0.3	87.0	Cook before serving /Ready to heat.
		Red rice	25	1.9	0.3	86.3	
		Groundnut	30	7.6	11.9	170.1	
		Soya oil	20	0.0	20.0	180.0	
		Total	100	15.6	32.5	523.4	
C.3	Sabudana khichdi	Groundnut	55	13.92	21.89	311.85	Cook before serving /Ready to heat.
		Sabudana	35	0.07	0.07	122.8	
		Soya oil	10	0	10	90	
		Total	100	13.99	31.96	524.65	

Supplementary Table 2 (S2) : Mineral Mix (MN)

Ingredients and nutritional information of MAHAN vitamin-mineral mix powder given as micronutrient (MN) supplements along with MAHAN LTFs.

**Nutritional information**			
Per 5 g provides approx.			
Energy	7.5 Kcal	D-Panthenol	3 mg
Carbohydrate	1.95 g	Vitamin A	1 mg
Fat	0 mg	Vitamin D3	800 IU
Calcium	350 mg	Vitamin E	20 mg
Phosphorus	181 mg	Vitamin K3	25 mcg
Magnesium	80 mg	Vitamin B1	0.5 mg
Potassium	50 mg	Vitamin B2	1.5 mg
Zinc	11 mg	Vitamin B6	0.6 mg
Iron	9 mg	Vitamin B12	1.6 mcg
Copper	1.4 mg	Vitamin C	50 mg
Iodine	100 mcg	Folic Acid	200 mcg
Selenium	20 mcg	Biotin	60 mcg
Nicotinamide	5 mg		
**Ingredients**: Sugar, Citric acid, orange flavour, sunset yellow, FCF, CMC Sodium, Cremophor RH 40, Aspartame.			

Each 100gms packet of MAHAN LTF along with 5gms of MN is equivalent to WHO RUTF package of 100gms in micro and macro nutrients. Each 100gms of WHO RUTF contains 13-16gms proteins, 520-550 calories, which are ready to use pack with micronutrients

Supplementary Table 3 (S3): Amount of LTF-MN to be fed to SUW according to weight

Weight (Kg)	3-4.9 kg	5-6.9 kg	7-9.9 kg	10-14.9 kg	15-20 kg
	Quantity of MAHAN LTF				
8 am	1/3 packet	1/2 packet	¾ packet	1.25 packet	1.5 packet
11 am	1/3 packet	1/2 packet	¾ packet	1.25 packet	1.5 packet
2 pm	1/3 packet	1/2 packet	¾ packet	1.25 packet	1.5 packet
5 pm	1/3 packet	1/2 packet	¾ packet	1.25 packet	1.5 packet
MAHAN Vit-Min mix was given with 100 gm of LTF.	3.125 gm	5 gm	5 gm	6.25 gm four	7.5 gm

Note: Ready-to-cook LTF dishes should be freshly cooked by VHW at her home.

For details of LTF, please refer our published paper: DOI: 10.29328/journal.afns.1001034

Ashish Satav* , Vibhawari Dani, Jayashri Pendharkar, Dipti Jain, Satav K. Mapping of Local Therapeutic Foods (LTF) and Micronutrients (MN); their logistics in community-based management of Severe Malnutrition (SAM, SUW) as a benchmark in tribal Melghat, Maharashtra ARCHIVES OF FOOD AND NUTRITIONAL SCIENCE. 2022:26-34

Supplementary Table 4 (Table S4): Important differences between Ready to use therapeutic food (RUTF) and local therapeutic food (LTF).

S. No.	RUTF for treatment of severe malnutrition.	LTF (Local Therapeutic Food) innovated by MAHAN.
1	Recovery rate of SUW after RUTF is <30%.[1, 2]	Recovery rate of SUW is 36% to 78.2%
2	Case fatality rate (CFR) after RUTF is 1 to 5%.[1-3]Most of these studies have treated uncomplicated SUW.	Case fatality rate is 0.29% for SUW who were treated with LTF during the 3 months of treatment period. We have treated both complicated and uncomplicated SUW with LTF.
3	RUTF is single dish and hence acceptance rate is very less. (40%)[4]	LTF are multiple, palatable, culturally acceptable dishes. Hence acceptance rate is >93%.[5]
4	RUTF is costly. (Rs. 26.80 Rs. per 92 gm)[6]	LTF is cost-effective (11-14 Rs. per 100 gm).
5	RUTF distribution at home leads to sharing of food by other siblings.[7] Hence it is not completely consumed by the SUW children. It leads to unbearable recovery rate of severely malnourished children.	LTF is distributed and fed by VHW in their presence in house of VHW. Hence no sharing of LTF in family.[5]
6	RUTF are industrial food benefitting industry and foreign food for people who lose confidence in home food.	LTF is prepared by local tribal females and hence give employment to tribal females.[8] LTF is prepared from local produce in a socio-culturally accepted way by local women in the form of various palatable dishes. It builds confidence in the local community that SUW can be treated with their own food.[5]
7	Not sustainable	Sustainable and replicable.

Supplementary File S7: Medication administered by VHW (As per WHO protocols)

<u>All SUW children were given:</u>

1. Folic Acid 5mg at the time of enrollment
2. Syrup Albendazole 400 mg is given in two doses-usually to children above the age of 1 year.
   i. 1st dose- after 14 days of enrollment
   ii. 2nd dose- after 15 days of 1st dose.

   Dose:
   - Children less than 2 years- 5ml
   - Children above 2 years- 10 ml
3. Oral Vitamin A supplementation 3,00,000 IU every six months is administered by VHW if not taken by child through Anganwadi/ASHA

<u>All enrolled children from IA and CA were given:</u>

1. Treatment for ARI
2. Treatment for Diarrhea
3. Treatment for Fever
4. Treatment for Malaria

1. Treatment for ARI: Co-trimoxazole BD for 5-7 days or Amoxicillin

Age (months)	Syrup (ml) (5 ml=Trimethoprim 40mg)	Tablet (1=Trimethoprim 80mg)
1 - 2	2.5	1
2 -12	5	2
13- 60	7.5	3

Or

Weight	Amoxicillin dispersible tablet 250 mg
Less than 10kg	Morning half tablet
	Evening half tablet
10-20 kg	Morning one tablet
	Evening one tablet

More than 20kg	Morning two tablets
	Evening one tablet

2. Treatment for Diarrheal diseases

Age (months)	MAHAN ORS† -ml after each stool	‡Norfloxacin, Syrup(ml) BD for 3-5 days (20 mg per ml) (5.2-17.9 mg/kg/day)	§ Metronidazole, -syrup (ml) 8 hourly for 5-7 days (5ml=200 mg).<br>(20-30 mg/kg/day) (as per body weight)
1-12	75	1 to 2.5 ml	1 to 2 ml
13- 60	150	2.5 to 5 ml (or tab. Norfloxacin 100 mg half to 1 BD)	2 to 3 ml

†*MAHAN- ORS is a modified WHO formula, , with addition of nutmeg, , 28.12 mg and cardamom, **0.125gms**/ 45gm, which increased the palatability, acceptability and efficacy.*
‡***For** Diarrhea if continued after 24 hours of ORS, or with severe dehydration or fever, or dysentery treatment.*
§ *For dysentery treatment.*

If Norfloxacin was not available then Syp. Furazolidone/Furoxone is given to children with diarrhea (indications as mentioned above) or dysentery.

Age (months)	‡ Furoxone, Syrup(ml) QID for 3 days (5ml =50 mg)<br>(7 mg/kg/day) (as per body weight)
1-12	0.5 to 1.5 ml
13- 60	1.5 to 3 ml

3. Treatment of vomiting:

Age (months)	Domperidone 5mg (0.3 mg/kg given T.D.S. before meals as per need)
1-12	¼ to half Tab. thrice a day
13- 60	Half to 1 Tab. thrice a day

4. Treatment for Fever

Paracetamol in the case of fever (temperature more than 100° F), headache or limb pain of the patient, after drinking milk or after eating food.

**Age**	**Paracetamol syrup (5 ml = 125 milligram)** (Two to Three times a day as per body weight: 10-15 mg kg per dose)
New-born baby to 1month	1 to 1.5 ml
1month to 1 year	2 to 5 ml
1 year to 5 years	5 to 8 ml (¼th tablet of 500mg)

If the patient's condition has not recovered/ worsens/ becomes unconscious/has reduced or absent urine output, then refer the patient immediately to the hospital.

4. Treatment for Malaria: Chloroquine syrup (5ml=50 mg Chloroquine base)

Age (months)	Doses			
	First (ml)	After 6 hours (ml)	After 12 hours (ml)	After 12 hours (ml)
1 to 12	4 to 9	2 to 4.5	2 to 4.5	2 to 4.5
13 – 60	9 to 18	4.5 to 9	4.5 to 9	4.5 to 9

Syrup- chloroquine-(after eating food or drinking milk) (1 ml=10mg)

(First dose, 10mg per kg body weight, second dose 5 mg per kg body weight, third dose 5 mg per kg body weight, fourth dose 5 mg per kg body weight.)

Supplementary File S8_Behavior Change Communication (BCC) by VHW and supervisors.

BCC after 90 days of LTF-MN therapy to 60 months

MAHAN has developed various modules and audio-visuals to deliver the BCC or health education to the community according to the socio-cultural context of the Melghat.

BCC is given to all the children enrolled in the study from both intervention area (IA) and control area (CA) during the study period while the IA received intensive BCC in addition to this routine BCC which is described in separate Appendix 8.

Behavior Change Communication (BCC) of parents of all children (0-60 months of age): It is door to door and One to one or group health education. The various methods are:

1. **Nutrition health education** to the parents of 0-5 years of age group children- 30 times in the during first 5 years of a child- with the help of Poshan flip chart. (once in two months)
2. **Complimentary feeding education** to the parents of children of age group from 4 months to 2 years, with the help of MAHAN module on complimentary feeding. (once a month till the age of 2 years).
3. **Antenatal care (ANC) health education** to the pregnant women during every visit (10 visits in a one cycle of pregnancy)- 10 times- with the help of flip chart on antenatal care.
4. **High risk pregnancy health education** to the high-risk mothers identified by the medical supervisors during ANC- with the help of MAHAN module on high-risk pregnancy.
5. **Breastfeeding health education**- to the pregnant women and lactating mothers with the help of MAHAN module on breastfeeding by medical supervisors.
6. **Disease health education** (pneumonia, diarrhea, malaria, vitamin-A, anemia, skin rashes) to the parents of 0-5 years children- all children of the village should be covered within three months (33% of the monthly target)- with the help of HBCC health education modules. It is delivered by BCC supervisors. (once in 2 months till the age of 60 months).

1. **Nail cutting and hand washing** demonstrations education to the parents of 0-5 years age children- all children of the village should be covered within three months (33% of the monthly target) by BCC supervisors. (once a weekly till the age of 60 months).

7. **Community Growth Chart:** The BCC supervisors during each village visit deliver health education to the parents of children in the age group of 0-5 years with the help of big growth chart. Children of different malnutrition categories are asked to sit on the growth chart and the parents are encouraged to start discussion among themselves for positive deviation. (once in a month)
8. **Street plays:** The supervisors and VHWs conduct regular (once in 4 months) street plays on various nutritional and health issues.

9. **Audiovisual (AV) film:** MAHAN has prepared an AV film in local dialect on nutrition, malnutrition and child deaths. It is shown once in 4 months to villagers and parents of SUW.
10. **Flip chart:** MAHAN has developed pictorial flipchart based upon photographs of Melghat community only. It is shown to the beneficiaries (parents of SUW) and other parents. Each parent receives this education at least 30 times in 5 years by VHWs and supervisors.
11. **Mass Community awareness programs.** During mass awareness program, once in 4 months, we arranged community meetings or gramsabha. During these meetings, more than 50% of the adults and key persons e.g. gaon panchayat members are present. Various health and nutrition issues are discussed and health and nutrition messages are exchanged with community. Various programs like Prabhat Feri, cleanliness drive, meeting with adults, sports for children, audiovisual shows, one to one counseling, group counselling, etc. were conducted during the programs.

Intensive Behavior Change Communication (I-BCC) for SUW children during SAMMAN intervention.

1. **Nutrition health education** to the parents of SUW children- weekly till 90 days of LTF-MN therapy- with the help of Poshan flip chart.
2. **Complimentary feeding education** to the parents of SUW children (age group from 6 months to 2 years)- weekly till 90 days of LTF-MN therapy - with the help of MAHAN module on complimentary feeding.
3. **Disease health education** (pneumonia, diarrhea, malaria, vitamin-A, anemia, skin rashes) to the parents of SUW children- weekly till 90 days of LTF-MN therapy - with the help of health education modules.
4. Demonstration of weekly n**ail cutting and daily hand washing of SUW children and** education of their parents till 90 days of LTF-MN therapy.
5. **Community Growth Chart:** The BCC supervisors during each village visit deliver health education to the parents of children in the age group of 0-5 years with the help of big growth chart. Children of different malnutrition categories are asked to sit on the growth chart and the parents are encouraged to start discussion among themselves for positive deviation. (once a week)

Supplementary File S9: Outcome indicators.

The definitions of SUW(WAZ ≤ -3SD), MUW (WAZ >-3SD and <-2SD of the median) and Normal (WAZ> -2SD of the median)followed WHO criteria.(1)(2)

Outcome selection by public patient involvement (PPI). After intensive community engagements and meetings, and community research projects (1998 to 2010),(3) we realized that the high prevalence and mortality of chronic malnutrition like SUW was a health priority of the community. Hence, we kept recovery rate, growth pattern, mortality, prevalence and relapse as the primary outcome measures, and episodes of infections as secondary outcome.

Outcome at 60 months of age in the LTF-MN group:

a) Non-recovery from SUW: If the status of a child remained SUW throughout the observation period.

Recovery from SUW:

Total no. of SUW cases who become normal or MUW after our intervention*100

Total no. of enrolled SUW cases.

b) Recovered without relapse: If the status of the SUW child changed to MUW/normal after the 90-day intervention and continued to have the same status till the end of the observation period.

c) Recovered from SUW with relapse: If the SUW child has at least two MUW/normal occurrences, with intermittent SUW occurrences. for the majority of time with MUW/normal occurrences, but the last status was SUW.

Outcome at 60 months of age in the control group: development of SUW or the child remains normal during the observation period.

d) Growth trajectory for weight for age of children: As per WHO child growth standards, growth pattern means growth velocity based on weight for age of a child from 6 to 60 months of age.

e) Case fatality rate (CFR) of SUW: It is the percentage of children with SUW who died during the study period (From January 2011 to October 2023).

CFR: the proportion of children who die from a disease contracted during a specific period of time, calculated by dividing the total number of deaths by the total number of cases and typically expressed as a percentage.

(Number of notified deaths / numbers of notified cases) *100 (Reference: THE GLOBAL HEALTH OBSERVATORY, WHO) https://www.who.int/data/gho/indicator-metadata-registry/imr-details/2445

CFR= Number of SUW children who died in one year *100/ Number of SUW children in one year.

f) Point prevalence of SUW: It the percentage of SUW children in the age group of 6 to 60 months out of total child population in that age group in the study area during a particular point of time (July to Sept) of each year.

Point prevalence of SUW =

$$\frac{\text{Total no. of SUW children (6-60 months) during one month} \quad \text{X 100}}{\text{Total no. children (in the age group of 6-60 months) in that population in one month.}}$$

g) Episodes of infections: Minimum interval between two episodes of fever, diarrhea, or ARI was considered as 7 consecutive days. Fever was considered if temperature was ≥99.5 °F.

h) Re-feeding diarrhea: Passing loose stools without blood or mucous after feeding LTF due to gastrocolic reflex, not associated with dehydration or weight loss.

Evaluation of project:

The project was evaluated by expert third party (medical colleges and university) for our outcome indicators.

1. Yang H, de Onis M. Algorithms for converting estimates of child malnutrition based on the NCHS reference into estimates based on the WHO Child Growth Standards. BMC pediatrics. 2008;8:19.

2. de Onis M, Onyango A, Borghi E, Siyam A, Blössner M, Lutter C. Worldwide implementation of the WHO Child Growth Standards. Public health nutrition. 2012;15(9):1603-10.
3. Satav AR, Satav KA, Bharadwaj A, Pendharkar J, Dani V, Ughade S, et al. Effect of home-based childcare on childhood mortality in rural Maharashtra, India: a cluster randomised controlled trial. BMJ Glob Health. 2022;7(7).

Supplementary File S10: Statistical analysis

The demographic and anthropometric characteristics of children were summarized according to the measurement scale. The wealth index for each child was obtained based on the household assets, using *categorical principal component analysis*. The first three principal components that captured major variability in the data were considered and the L_3norm score was obtained for each child, as an index of the wealth status of a child. The scores were partitioned into quartiles, with the lowest quartile indicating poor status, and the highest quartile indicating good wealth status. On similar lines, the village-level categorization was performed by referring to village-level attributes. The *weight for age* Z-score was obtained for each child using the WHO Child Growth Reference (2006) (4) or the cohort of children, at each follow-up time. The R-library *Z scorer*, which has an inbuilt WHO Growth Reference data, was used for the computations. For each child, a categorical assessment, as per the operational definition, was obtained till the age of 60 months, or earlier in case of loss to follow-up. The comparison of the change in Z-scores from baseline to 3 months and baseline to 60 months of child age (last observation), between the LTF-MN and control groups was performed as per the age of enrolment categories. For baseline to 3 months, the estimated marginal mean change in Z-scores for the 2 groups was obtained using analysis of covariance (ANCOVA), considering baseline age as a covariate within the category. Similar analysis was followed for baseline to 60 months. Fixed factors like sex, birth weight, parent literacy, community type, wealth index, and village-level facilities were retained in both analyses.

For the LTF-MN group, the dichotomous outcomes 'recovered' or 'not recovered' were considered, and the effect of fixed factors on the outcome at 3 months and 60 months of age, was determined through *multiple logistic regression*. The effect of factors on the 'recovery with relapse' was obtained considering 'recovery without relapse' as a reference through *multiple logistic regression*. The significant village-level attributes contributing to 'recovery without relapse' were determined by referring to *Wald's statistic*s.

Supplementary File S11: Patient and public involvement (PPI)

Box 1: Patient and public involvement in GRIPP2 according to BMJ guidance

1) How was the development of the research question and outcome measures informed by patients' priorities, experience, and preferences?

Reply: From 1998 to 2004, we conducted multiple interviews, meetings and focus group discussions with the local tribal community members, parents of patients of severe malnutrition /underweight (SUW) of Melghat, service providers, social workers and local community-based organizations. We also treated patients in the community during that period. Our local tribal counselors in all government hospitals also conducted multiple FGDs and interviews with the parents of patients and community members to understand the research need. Because of above PPI, we realized that severe malnutrition /SUW and its deaths are major concern of the community. Based on these inputs from those stakeholders, we conducted systematic research from 2004 to 2010 and observed a very high prevalence of severe malnutrition, particularly SUW (18.7%)[1] and deaths[1, 2] in Melghat. This raised questions about the alignment of existing government health care policies with the community's actual needs, low priorities of government and international agencies about SUW, awareness of stakeholders about low health seeking behavior and under reporting of SUW. Additionally, prevalent socio-cultural practices seemed to contribute to low health-care seeking behavior. In response, we conducted a survey to delve into the root causes of the high prevalence of SUW in Melghat. To conduct the research and surveys, as per the need of the local community we involved local semiliterate tribal females selected by the local tribal community members during community meetings (gramsabha). All these inputs were used for development of the research questions and outcome measures related to SUW.

2) How did you involve patients in the design of this study?

Reply: We involved both community and parents of patients as research partners in all aspects of the study including identifying the original research question, importance of SUW and need for its treatment. We also involved them for deciding the need and study design of community-based care of severe malnutrition, and especially treatment by local semiliterate social service minded married tribal women as village health workers (VHWs). The community members and parents of patients with our team members selected the VHWs during gramsabhas.

To address local issues, we integrated community participation and Patient and Public Involvement (PPI) into our intervention, utilizing gramsabhas (community meetings), door-to-door surveys, and focus group discussions.

3) Were patients involved in the recruitment to and conduct of the study?

Reply: The parents of patients and the local community members, along with community-based organizations and local service providers helped for the recruitment of participants for the trial. They helped to pilot the community-based survey for the first phase of the trial which has improved the response rate. They also worked with the researchers to collate comments from pilot survey, to adapt methods, and to feed back to the participants for the next trial. They checked comprehension of changed items and comments from the lay perspective. They took part in the consensus gramsabhas, to agree consensus on solutions and interventions for SUW management.

4) How will the results be disseminated to study participants?

Reply: The results of the trial were discussed with the community members and the parents of the l study participants during gramsabhas. The authors will disseminate the results via conference presentations and publications in national and international journals. Funding bodies will be informed about the results via email and government machinery will be informed via email and during advocacy meeting.

1. Dani V, Satav A, Pendharkar J, Ughade S, Jain D, Adhav A, et al. Prevalence of under nutrition in under-five tribal children of Melghat: A community based cross sectional study in Central India. Clinical Epidemiology and Global Health. 2015;3(2):77-84.

2. Satav AR, Satav KA, Bharadwaj A, Pendharkar J, Dani V, Ughade S, et al. Effect of home-based childcare on childhood mortality in rural Maharashtra, India: a cluster randomised controlled trial. BMJ Glob Health. 2022;7(7).

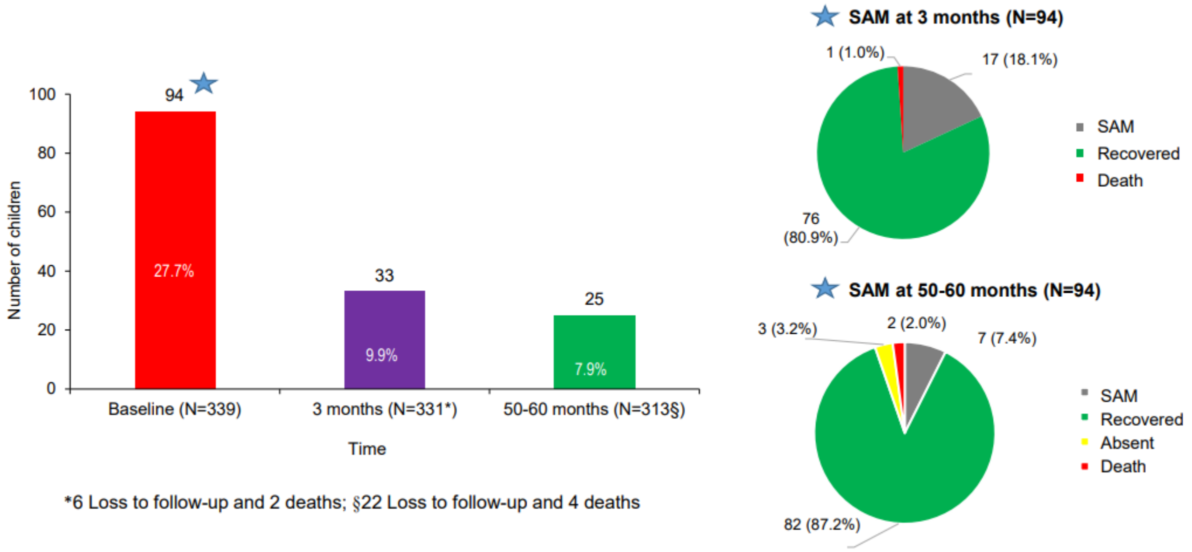

Supplementary Figure 1: Proportion of SAM out of SUW at three time points.

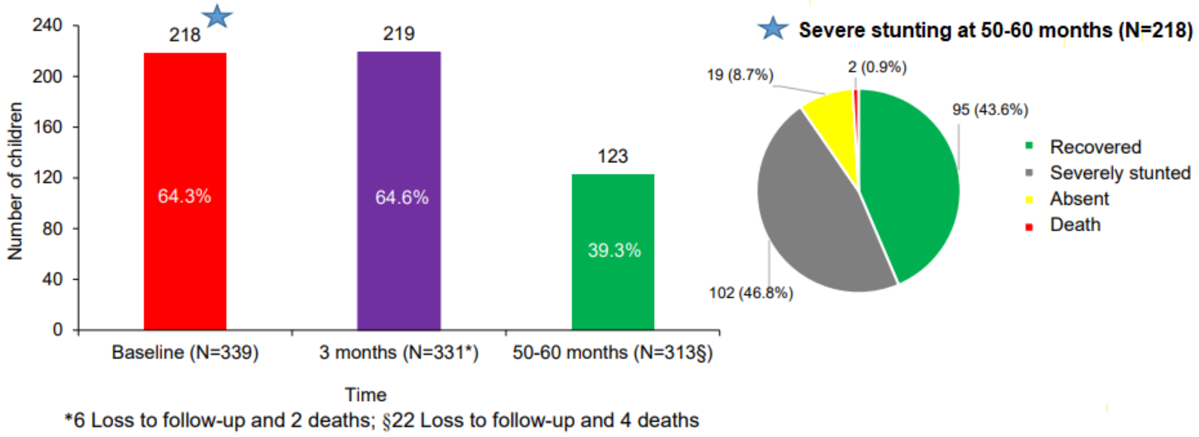

Supplementary Figure 2: Proportion of stunting out of SUW at three time points.

Supplementary File S12- Comparison of Studies with community based management of undernutrition and SUW.

Table A: Methodology

Reference<br>Author<br>Country<br>Year	Study period<br><br>Study design	Participants	Setting	Intervention delivered by<br><br>Duration	Data for SUW	Intervention			Complicated children
						Food supplementation	Treatment of Infections	BCC	
(9)<br>Hossain et al.<br>Bangladesh.<br>2011	June 2005<br>to<br>June 2007<br><br>Randomized, non-masked intervention trial	507 SUW children aged 6-24 months who were post-diarrheal discharged from hospital	Hospital as well as community	Hospital staff<br>Female Health Workers (HWs)<br><br>3 Months	Primary data	1.Hospital control group (Gp I): HE and micronutrients and growth monitoring every two weeks at HNFU<br>2.Community-based follow-up (Gp II): HE and micronutrients and growth monitoring every two weeks at CNFU<br>3.Community-based follow-up plus supplementary food (Gp III): HE and micronutrients and growth monitoring every two weeks at CNFU<br>Supplementary food: a)6-12 months-6 gm protein and 150Kcal<br>b)12-24 months- 12gm protein and 300Kcal<br>Sibling sharing was avoided by providing extra packets.<br>4.Community-based follow-up plus PS (Gp IV): HE and micronutrients and growth monitoring every two weeks at CNFU<br>Psychosocial stimulus<br>No supplementary food<br>5.Community-based follow-up plus SF and PS (Gp V): HE and micronutrients and growth monitoring every two weeks at CNFU<br>Supplementary food<br>Psychosocial stimulus	No antibiotics after assigning the group.<br><br>Chilldren received:<br>Immunization, deworming	Health Education	Excluded

(2)<br>Odei Obeng et al.<br>2022	Pooled secondary data analysis of SUW from primary data for SAM Between 2010 to 2020<br>Total 13 studies-11 RCTs and 2 CMAM programs.	24,829 children with 55% SUW aged 6–59 months	13 datasets. from 9 countries (2 Asia, 6 Africa and 1 Middle east country)	-	Secondary data	Three groups:<br>1. low-intensity T/t (20.3%): home foods or fortified blended flours.<br>2. mid-intensity T/t (23.9%):<br>LNS-75 kcal/kg/day. (~500Kcal)<br>3. high intensity T/t (55.8%)-<br>LNS- 175 kcal/kg/day.(~1000 Kcal)<br>LNS includes RUTF and RUSF.	NA	NA	NA
(10)<br>S. Kumar, L. Bhawani. Baran, Rajsthan, India.<br>2005	March 2003 to August 2003<br><br>Single arm study, feedings partially supervised.	MAM with 27.3% prevalence were the study participants.<br>But prevalence of SUW before and after is the outcome indicator	Community intervention in tribal region	Anganwadi Workers, Health workers and volunteers<br><br>Six months	Indirect assessment of SUW<br><br>Interventio n given to MAM children	500-700 Kcal/day in 160gm of food divided in 5 feeds as supplementary feeding:<br>Supervised feeds 3 times at NCC, THR for 2 times	No antibiotics<br><br>ORS given for diarrhea.<br>Vitamin A<br>Deworming	Health education given<br>IEC done	Excluded
(1)<br>Bailey et al. Kenya and South Sudan.<br>2021	May 2017 to August 2018<br>Cluster randomized non-inferiority trial (With 12 clusters in Kenya and 12 clusters in South Sudan)	416 SUW children receiving standard therapy in Group 3	Partial rural and urban.<br>Partial health Facility and community	Hospital staff<br>Community health workers<br><br>17 weeks	Secondary data	RUTF therapy as per WHO norms	No	No	Excluded

(11)<br>Kundan et al.<br>Nandurbar, Maharashtra, India.<br>2021	June 2014 to July 2016.<br><br>Secondary data analysis for SUW Children.<br>Primary study of SAM children.	5979 SAM children aged 6-59 months	Community intervention in tribal region	CHW and Anganwadi Workers<br><br>Eight weeks	Secondary data	Three groups:<br>1. RUTF with micronutrients- commercial<br>2. RUTF with micronutrients- locally prepared<br>3. Amylase rich food with micronutrients (Sprouted grams and wheat)<br>Take Home Ration (THR) given<br>Home food allowed	No	No	Excluded
(12)<br>Thakwalakwa et al.<br>Malawi.<br>2011	Enrolment: November 2007 to January 2008.<br>Take Home Ration supplementary therapy for 12 weeks<br>Randomised, controlled, assessor-blinded clinical trial	299 children <-2 WAZ of 6-15 months age	Community intervention at rural region	Take Home Ration by Health Centers<br><br>12 weeks	Primary data	Gp I (n-86)- no supplements.<br>Gp II- corn-soya mix (n-109)- THR 2kg every 4 weeks with 70gm/day (280Kcal)<br>Gp III- lipid based nutrient supplement (n-104)- THR 1.2 kg every 4 weeks with 43 gm/day (220 Kcal)<br>Micronutrient added in different strengths<br>Breastfeeding continued	No	No	Excluded
(13)<br>Hossain et al.<br>Bangladesh.<br>2005	Cross sectional study 2002	2388 children 6-23 months<br><br>6815 children 6-59 months	Project hoouseholds-4539<br>Non project households-2276	Health workers<br><br>6 years duration	Primary data	Bangladesh Integrated Nutrition Program (1995-2002) already done.<br>Assessed for the prevalence of nutritional status (WAZ, WHZ, HAZ).	-	-	NA
(3)<br>Ciliberto et al.<br>Malawi.<br>2004	Dec 2002 to June 2003<br>A controlled, comparative, clinical	1178 children WHZ<-2 aged 10–60 months	Uraban and rural Nutritional Rehabilitation Units	Hospital staff<br><br>8 weeks duration	No data for SUW	Two groups:<br>1. Standard therapy (186 children)<br>2. Home-based therapy with RUTF (992 children).<br>Take home ration given as a soul food.	No	No	Excluded

	effectiveness trial (ST:RUTF= 1:4)								

Table B: Results

Author Country Year	Recovery at the end of nutritional therapy	Weight gain g/kg/day	Lost to follow up during therapy	Post-therapy Follow up duration	Relapses	Episodes of Infections	Recovery at the end of follow-up	Community prevalence	CFR
(9)<br>Hossain et al. Bangladesh. 2011	Gp I=24%,<br>Gp II=29%,<br>**Gp III=34%,**<br>Gp IV=40%,<br>**Gp V=53%.**	Median wight gain after 12 weeks T/t:<br><br>Gp III, V= 0.88-1.01 kg,<br><br>Gp I, II= 0.63–0.76 kg	Dropped-out: Gp I=29.4%, Gp II=24.2%, **Gp III=13.9%,** Gp IV=34.3%, **Gp V=26.2%**	No follow up.	NA	During treatment 8.08%	NA	No community prevalence seen	Gp I- 0.98%<br><br>However defaulter rate high
(2)<br>Odei Obeng et al. 2022	SUW+SAM+MAM= 28.3%<br>~500Kcal=38.8%<br>~1000 Kcal=28.3%<br>Recovery criterias:<br>MUAC ≥12.5<br>WHZ ≥-2<br>no oedema	SUW+SAM=4.14<br><br>SUW+SAM+Oedema=4.26<br><br>SUW+MAM=3.55	SUW Defaulters= 9.4%	NA	NA	In-patient transfer=6.2%	NA	Prevalence of WAZ <-3 among children admitted to CMAM- 55%	SUW+SAM= 2.4%<br><br>SUW+MAM=0.64%
(10)<br>S. Kumar, L. Bhawani. Baran Rajsthan, India. 2005	Only SUW prevalence studied before and after	NA	NA	No follow up	NA	Initial survey. Recall for last 15 days (March 2003):<br><br>Fever 29%, Cough 25%, Diarrhea 8%	NA	Initial prevalence. March 2003: SUW= 32.9%, Prevalence at the end.	NA

								Septeber 2003: SUW= 26.1%	
**Author Country Year**	**Recovery at the end of nutritional therapy**	**Weight gain g/kg/day**	**Lost to follow up during therapy**	**Post-therapy Follow up duration**	**Relapses**	**Episodes of Infections**	**Recovery at the end of follow-up**	**Communit y prevalence**	**CFR**
(1) Bailey et al. Kenya and South Sudan. 2021	Standard Therapy: Group 3=13.7% (recovery from SAM)	NA	During therapy: 45.2%	Standard therapy: 6 clusters each from Kenya, Sudan<br>Only Kenya had 4 months follow up<br>Total loss-to follow-up in all groups- 60%	8% relapse of SAM in Group 3 of Standard therapy Kenya	During therapy not mentioned	Kenya std th. Gp3 .n-26 .recovered not mentioned .non recovered: 5 (19%)	NA	CFR During therapy<br><br>Group 3 Standard therapy: 1.7%
(11) Kundan et al. Nandurbar, Maharashtra, India. 2021	After 8 weeks: SAM+SUW+Severe Stunted= 44.97%<br>SAM+SUW= 34.20%	After 8 weeks: SAM+SUW+Severe Stunted= 2.720<br>SAM+SUW= 2.235	NA	No follow up	NA	NA	NA	NA	NA

(12) Thakwalakwa et al. Malawi. 2011	NA	Mean weight gain after 12 wk therapy: Control= 630gm; Gp I=680gm; Gp II=750gm P value-0.211	Lost to follow up: Control= 7% Gp I= 0.9% Gp II=2.8%	Follow up not done	NA	NA	NA	Community prevalence not seen	Control=3.5%. Gp I=1.8%. Gp II=1.9%
(13) Hossain et al. Bangladesh. 2005	NA	NA	NA	NA	NA	NA	NA	SUW: Project= 11.4% Non-project= 12.1%; MUW: Project= 35.2% Non-project= 36.3%	NA
(3)Ciliberto et al. Malawi. 2004	ST=49% RUTF=72%	ST=3.0±8.8, RUTF=3.7±4.3.	ST: 8% RUTF: 9.9%	6 months	ST= 39.1%, RUTF=2.2%	NA	NA	NA	ST=6.2% RUTF= 3.7%

References: (Appendices other than old Appendix 11)

1. Hossain MI, Nahar B, Hamadani JD, Ahmed T, Brown KH. Effects of community-based follow-up care in managing severely underweight children. J Pediatr Gastroenterol Nutr. 2011;53(3):310-9.
2. Odei Obeng-Amoako GA, Stobaugh H, Wrottesley SV, Khara T, Binns P, Trehan I, et al. How do children with severe underweight and wasting respond to treatment? A pooled secondary data analysis to inform future intervention studies. Matern Child Nutr. 2023;19(1):e13434.
3. Kumar S, Bhawani L. Managing child malnutrition in a drought affected district of Rajasthan--a case study. Indian J Public Health. 2005;49(4):198-206.
4. Bailey J, Lelijveld N, Khara T, Dolan C, Stobaugh H, Sadler K, et al. Response to Malnutrition Treatment in Low Weight-for-Age Children: Secondary Analyses of Children 6-59 Months in the ComPAS Cluster Randomized Controlled Trial. Nutrients. 2021;13(4).
5. Kundan I, Nair R, Kulkarni S, Deshpande A, Jotkar R, Phadke M. Assessment, outcomes and implications of multiple anthropometric deficits in children. BMJ Nutr Prev Health. 2021;4(1):267-74.
6. Thakwalakwa C, Ashorn P, Phuka J, Cheung YB, Briend A, Puumalainen T, et al. A lipid-based nutrient supplement but not corn-soy blend modestly increases weight gain among 6- to 18-month-old moderately underweight children in rural Malawi. J Nutr. 2010;140(11):2008-13.
7. Hossain SM, Duffield A, Taylor A. An evaluation of the impact of a US$60 million nutrition programme in Bangladesh. Health Policy Plan. 2005;20(1):35-40.
8. Ciliberto MA, Sandige H, Ndekha MJ, Ashorn P, Briend A, Ciliberto HM, et al. Comparison of home-based therapy with ready-to-use therapeutic food with standard therapy in the treatment of malnourished Malawian children: a controlled, clinical effectiveness trial. Am J Clin Nutr. 2005;81(4):864-70.

References: (For old Appendix 11: **Comparison of Studies with community based management of undernutrition and SUW.**

1. Bailey J, Lelijveld N, Khara T, Dolan C, Stobaugh H, Sadler K, et al. Response to Malnutrition Treatment in Low Weight-for-Age Children: Secondary Analyses of Children 6-59 Months in the ComPAS Cluster Randomized Controlled Trial. Nutrients. 2021;13(4).
2. Odei Obeng-Amoako GA, Stobaugh H, Wrottesley SV, Khara T, Binns P, Trehan I, et al. How do children with severe underweight and wasting respond to treatment? A pooled secondary data analysis to inform future intervention studies. Maternal & child nutrition. 2023;19(1):e13434.
3. Ciliberto MA, Sandige H, Ndekha MJ, Ashorn P, Briend A, Ciliberto HM, et al. Comparison of home-based therapy with ready-to-use therapeutic food with standard therapy in

the treatment of malnourished Malawian children: a controlled, clinical effectiveness trial. Am J Clin Nutr. 2005;81(4):864-70.
4. Ali E, Zachariah R, Dahmane A, Van den Boogaard W, Shams Z, Akter T, et al. Peanut-based ready-to-use therapeutic food: acceptability among malnourished children and community workers in Bangladesh. Public Health Action. 2013;3(2):128-35.
5. Dani V, Satav K, Pendharkar J, Satav A, Ughade S, Adhav A, et al. Community-based management of severe malnutrition: SAM and SUW in the tribal area of Melghat, Maharashtra, India. Clinical Epidemiology and Global Health. 2017;5(2):62-9.
6. Compliance Report of Women and Child Development Department Public Interest Litigation No. 133 of 2007 PIL(2022).
7. Kabalo MY. Manifolds boosting severe acute malnutrition burden among children in and around Wolaita Zone, Southern Ethiopia: mini-review. BMC Res Notes. 2018;11(1):870.
8. Owino VO, Irena AH, Dibari F, Collins S. Development and acceptability of a novel milk-free soybean-maize-sorghum ready-to-use therapeutic food (SMS-RUTF) based on industrial extrusion cooking process. Maternal & child nutrition. 2014;10(1):126-34.
9. Hossain MI, Nahar B, Hamadani JD, Ahmed T, Brown KH. Effects of community-based follow-up care in managing severely underweight children. J Pediatr Gastroenterol Nutr. 2011;53(3):310-9.
10. Kumar S, Bhawani L. Managing child malnutrition in a drought affected district of Rajasthan--a case study. Indian J Public Health. 2005;49(4):198-206.
11. Kundan I, Nair R, Kulkarni S, Deshpande A, Jotkar R, Phadke M. Assessment, outcomes and implications of multiple anthropometric deficits in children. BMJ Nutr Prev Health. 2021;4(1):267-74.
12. Thakwalakwa C, Ashorn P, Phuka J, Cheung YB, Briend A, Puumalainen T, et al. A lipid-based nutrient supplement but not corn-soy blend modestly increases weight gain among 6- to 18-month-old moderately underweight children in rural Malawi. J Nutr. 2010;140(11):2008-13.
13. Hossain SM, Duffield A, Taylor A. An evaluation of the impact of a US$60 million nutrition programme in Bangladesh. Health Policy Plan. 2005;20(1):35-40.
